# Energy Expenditure, Dietary Energy Intake, and Nutritional Supplements in Adolescent Volleyball Athletes versus Nonathletic Controls

**DOI:** 10.3390/nu15071788

**Published:** 2023-04-06

**Authors:** Madison Bell, Ravneet Ghatora, Maria Ilektra Retsidou, Efthalia (Elia) Chatzigianni, Panagiota Klentrou

**Affiliations:** 1 Department of Kinesiology, Brock University, St. Catharines, ON L2S 3A1, Canada; mb14pf@brocku.ca (M.B.); rg19ym@brocku.ca (R.G.); ir22sf@brocku.ca (M.I.R.); 2 Department of Sport Management and Organization, University of Peloponnese, 23100 Sparta, Greece; echatzi@uop.gr

**Keywords:** athletes, adolescents, body mass, energy expenditure, caloric intake, macronutrient intake, micronutrient intake

## Abstract

Evidence suggests that athletes competing in team sports do not follow dietary recommendations. However, only few studies have investigated energy needs and supplement use in adolescent athletes, and whether they are meeting their energy requirements. This observational study examined energy expenditure, dietary energy intake, and use of nutritional supplements in 58 adolescent (14–17 years old) volleyball athletes (15 males, 43 females) and 58 age-matched nonathletic controls (13 males, 45 females). Participants completed an online survey including questions on demographic information, body mass, and a series of standardized questionnaires assessing energy expenditure, dietary energy, macronutrient, micronutrient, and supplement intake. Energy expenditure relative to body mass was higher in athletes than nonathletes by 13 kcal/kg/day (group effect, *p* < 0.001), and in males compared to females by 5.7 kcal/kg/day (sex effect, *p* = 0.004). Athletes had higher energy intake than nonathletes (+6.4 kcal/kg/day, *p* = 0.019) and greater consumption of fruits (*p* = 0.034), vegetables (*p* = 0.047), grains (*p* = 0.016), dairy (*p* = 0.038), meats and meat alternatives (*p* < 0.001), as well as higher intakes of fat (*p* < 0.001), carbohydrates, protein, sugar, fiber, vitamin C, calcium, and sodium (*p* = 0.05) compared to nonathletes. The average protein intakes exceeded the upper recommendations in all groups, suggesting that this is not a nutrient of concern for young volleyball athletes. However, athletes were only meeting 60% of the estimated energy requirements (EER) for their age, height, body mass, and physical activity score, (3322 ± 520 kcal/day), while nonathletes were meeting 74% of the EER (*p* < 0.001). The relative energy balance of male athletes was lower compared to both female athletes (*p* = 0.006) and male nonathletes (*p* = 0.004). Finally, more athletes reported using performance-related supplements than nonathletes, but there were no differences in the consumption of other dietary supplements. Overall, when compared to nonathletic controls, both male and female adolescent volleyball athletes were found to match their higher energy expenditure with a greater dietary energy intake; however, all adolescents were below the estimated energy requirements, a finding more profound among the volleyball athletes.

## 1. Introduction

Physical activity and sports participation are important determinants of adolescent health [1,2]. Volleyball is one of the most popular sports worldwide, and it is played by more than 200 million people of all ages [3]. It is considered a high-intensity sport that stimulates both the aerobic and anaerobic energy production pathways [4]. It is believed that both the training and the diet of young volleyball players can make a difference in their performance [5,6,7]. Indeed, young athletes must meet their daily energy, macronutrient, and micronutrient needs. An imbalance between energy expenditure and intake, where expenditure exceeds intake, can lead to a loss of muscle mass, decreased performance, or an increased risk of injury [8]. This is more important during adolescence because this is a period of a noticeable growth spurt, involving an accelerated accretion of muscle mass as high as 2.3 g/day and 3.8 g/day in females and males, respectively [9]. Thus, to meet both the performance and growth requirements, the daily energy intake of young volleyball athletes must meet, or slightly exceed, their daily energy expenditure. However, there is evidence that athletes competing in team sports, including volleyball, do not follow dietary recommendations [10,11,12]. Additionally, female athletes, although seeming to consume more protein and fats, have a total energy intake less than that of male athletes [5,13,14].

Moreover, the appropriate consumption of some nutritional supplements may enhance sports performance [15]. Such nutritional supplements include dietary supplements, sport nutrition products, and ergogenic substances [15]. In general, nutritional supplements seem to promote athletes’ health and some are used to enhance performance either via fuel substrate utilization or by supporting the immune system [16,17]. Among adolescents engaging in sports training, dietary supplement consumption has been shown to vary based on age and sex [18,19,20,21], as well as sport-specific variables, i.e., team versus individual sport and level of competition [21,22,23,24,25]. Younger adolescents have been found to consume supplements related to health (e.g., to improve immune function) while older adolescents consume supplements for performance reasons (e.g., for quick recovery, increased strength, and endurance) [20,21,22,23]. Differences between the sexes have also been reported, with males reporting increased rates of performance-enhancing supplements compared to the health-related supplements consumed by female athletes [24,25,26,27]. Furthermore, athletes involved in individual sports have been shown to consume more supplements than those engaged in team sports, maybe due to the competitive independence of these athletes and the different social support provided [24,28]. In addition, power/strength (e.g., football, rugby, powerlifting) and endurance type sports (e.g., cross country, cycling) report higher prevalence rates of dietary supplement consumption than any other sport types (e.g., aesthetic, technical, ball sports, contact), with some studies showing differences also in the type of supplements consumed [18,29,30,31], while others do not [20,21,32,33,34,35,36]. Thus, additional research has been recommended to further understand the use of nutritional supplements per sport.

The aim of this study was to examine the energy expenditure, dietary intake, and nutritional supplement consumption in adolescent volleyball athletes as compared to age-matched nonathletic controls. It was hypothesized that both athletes and nonathletes would not meet Canada’s recommended intake guidelines, and that although athletes would have a greater intake overall than nonathletes, it would not be enough to match their higher energy expenditure requirements. We also expected to find a higher consumption of dietary supplements among the athletes compared to the nonathletic controls.

## 2. Materials and Methods

### 2.1. Participants

A convenient sample of adolescent volleyball athletes and nonathletes were recruited online from across Ontario, Canada; athletes were recruited by invitation during Canada Games, and through volleyball clubs, while nonathletes were recruited through social media. Participants were invited to complete an online retrospective survey looking at energy expenditure, dietary energy and nutrient intake, and supplement use. A total of 116 participants completed the study, including 58 volleyball players (*n* = 15 male, *n* = 43 female) and 58 nonathletic controls (*n* = 13 male, *n* = 45 female). All participants obtained permission from a parent and or legal guardian and confirmed their eligibility, which was to be 14–18 years of age, and either a volleyball athlete (elite club player, training for a minimum 6 h/wk) or nonathlete (not participating in competitive sports). The study and all related procedures received ethical clearance from the Brock University Research Ethics Board.

### 2.2. Procedures and Assessments

This observational study used a cross-sectional design to examine the energy expenditure, dietary energy intake, and supplement use of adolescent volleyball athletes as compared to age-matched nonathletic controls. Participants were recruited through social media (nonathletes) and/or through club coaches (athletes) during the period of June 2022 to October 2022. Participants were contacted by email with a link to access the online consent/assent forms and anonymous survey. The study involved a two-part survey administered using Qualtrics (XM, Provo, UT, USA) to obtain information such as questions about age, sex, body mass and height, household income, and parent education. Part 2 had participants answer questions about their physical activity and diet over the past 6 months using a standardized questionnaire. All answers were anonymous, and the survey took an average of 30 min to complete. The same blinded researcher examined and analyzed the data. The reporting of the study conforms to the STROBE statement [37].

#### 2.2.1. Demographic and Physical Characteristics

Participants answered a range of questions about their age (years), height (cm), body mass (kg), and sex. In addition, athletes were asked questions regarding their training history (years in sport) and training volume (hours/week). Body mass index (BMI, kg/m^2^) was subsequently calculated using self-reported body mass and height [38]. Classifications of BMI are made by weight class using the BMI range: underweight (<18.5 kg/m^2^), normal (18.5–24.9 kg/m^2^), overweight (25–29.9 kg/m^2^), and obesity (>30 kg/m^2^) [38].

#### 2.2.2. Food Frequency and Activity Questionnaire (FFAQ)

The Block 2014 FFAQ (NutritionQuest, Berkeley, CA, USA) was used to assess energy intake, diet quality, and physical activity over the past six months. This FFAQ is a commonly used measure of dietary intake and quality and energy expenditure [39], and has been tested for reliability and validity in Canadian women [40]. It includes 127 food and beverage items and additional questions to determine fat, protein, carbohydrate, sugar, and other food group serving consumptions. The participants were given a portion size sheet to help them quantify the amounts of food that they were eating and to better aid them in answering the questions. Dietary habits were examined with a focus on estimating the consumption of macronutrients (carbohydrates, fats, proteins), micronutrients (vitamins and minerals), and overall mean energy intake in kcal/day [41]. The FFAQ also provided estimated daily servings for Health Canada’s five main food groups, including grains, meats, meat alternatives, fruits, and vegetables, which were also compared against Canada’s Food Guide regarding servings per day [42].

The physical activity section listed various activities to collect information on the frequency and duration of each activity on a weekly basis for the six months preceding the survey. This information was used to calculate the average number of metabolic equivalent (MET) minutes of light, moderate, and vigorous physical activity that the students engaged in per day, and the average daily energy expenditure (kcal/day). Subsequently, we compared energy intake requirements relative to the energy expenditure, age, height, and body mass using the Canadian estimated energy requirement (EER) equations [43]:

EER for boys 9–18 years old = 88.5 − (61.9 × age (years)) + PAL × [(26.7 × body mass (kg)) + (903 × height (m))] + 25

EER for girls 9–18 years old = 135.3 − (30.8 × age (years)) + PAL × [(10.0 × body mass (kg)) + (934 × height (m))] + 25

where PAL is the physical activity level, i.e., sedentary, low active, active, very active [43]. As per the Health Canada classification for 14–18-year olds, male athletes were assigned a PAL of 1.42 and female athletes were assigned a PAL of 1.56, while both male and female nonathletes were assigned a PAL of 1. Finally, energy balance was calculated as the difference between energy intake and energy expenditure, both absolute (kcal/day intake–kcal/day expenditure) and relative (kcal/kg/day intake–kcal/kg/day expenditure).

### 2.3. Statistical Analysis

Analyses were performed using SPSS (IBM SPSS Statistics, 2021). Statistical significance was set at *p* = 0.05. All variables were first checked for normality using the Kolmogorov–Smirnov test, as well as skewness and kurtosis of ±3. Outliers were assessed and adjusted to the group mean. No assumptions were violated. Two-Way ANOVAs were used to examine differences between sexes, groups (athletes versus nonathletes), and their interaction regarding energy intake, energy expenditure, macronutrients, and micronutrients, as well as diet components. If a significant sex-by-group interaction was detected, independent sample *t*-tests were used to evaluate group differences within each sex. Chi-square (*χ*^2^) tests were used to examine differences in the number of male and female athletes and nonathletes regarding the use of performance and dietary supplements.

## 3. Results

The demographic characteristics of the participating cohort are presented in Table 1.

The physical characteristics of the participants are presented in Table 2. A significant difference was found in the self-reported body mass between male and female athletes, but not between male and female nonathletes or between the athletes and their nonathletic counterparts (Table 2). Due to these differences, energy expenditure and energy intake were examined in absolute terms, as well as relative to body mass. There were no differences between sexes or groups in BMI.

**Figure 1:**
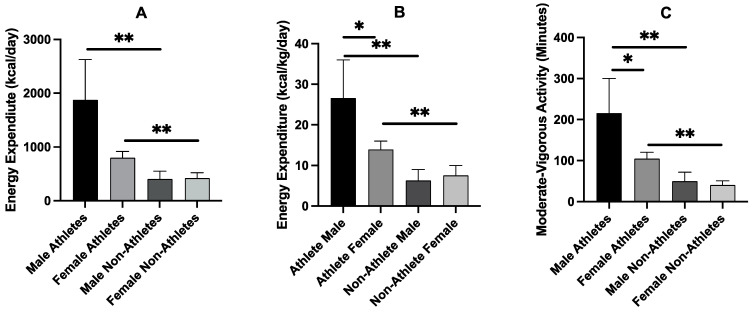
Absolute energy expenditure (**A**), relative energy expenditure (**B**), and moderate-vigorous activity (**C**) in male and female athletes (*n* = 13 and *n* = 45, respectively) and nonathletes (*n* = 13 and *n* = 45, respectively). Values are means ± SD. * denotes *p* < 0.05 difference; ** denotes *p* < 0.001 differences.

We found a significant sex-by-group interaction for both the absolute (kcal/day) (F = 18.8, *p* < 0.001) and relative (kcal/kg/day) (F = 13.2, *p* < 0.001) energy expenditure, as well as weekly minutes of moderate-vigorous activity (F = 12.4, *p* < 0.001). As shown in Figure 1, male athletes had higher energy expenditure than their nonathlete counterparts (1472 kcal/day difference, F = 20.4, *p* < 0.001 and 20.3 kcal/kg/d difference, F = 22.9, *p* < 0.001). Likewise, female athletes had higher energy expenditure than female nonathletes (377 kcal/day difference, F = 1.26, *p* < 0.001 and 6.3 kcal/kg/day difference, F = 1.65, *p* < 0.001). In addition, male athletes had greater energy expenditure than female athletes (12.7 kcal/kg/day difference, F = 31.4, *p* = 0.016). Differences were also found in weekly moderate-vigorous physical activity (Figure 1C) between male athletes and nonathletes (166 min/week difference, F = 14.5, *p* < 0.001), between female athletes and nonathletes (64 min/week difference, F = 5.4, *p* < 0.001), and between male athletes and their female counterparts (111 min/week difference, F = 30.4, *p* = 0.014).

**Figure 2:**
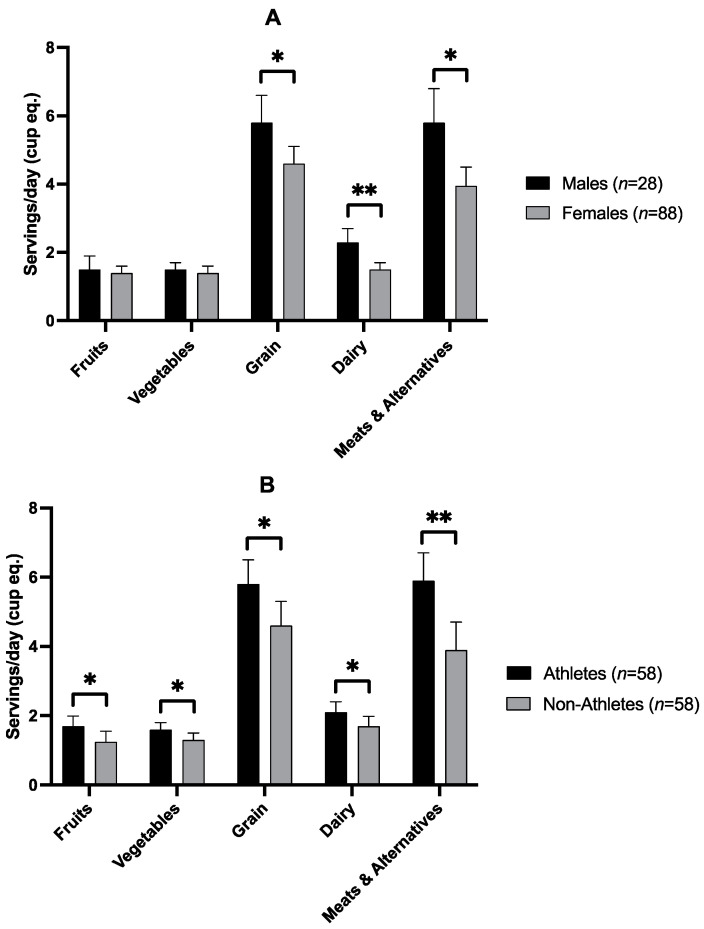
Servings per day for fruits, vegetables, grains, dairy, meat, and alternatives in males versus females (**A**), and athletes versus nonathletes (**B**). Values are means ± SD. * denotes *p* < 0.05 difference between sexes (*p* = 0.05); ** denotes *p* < 0.001 difference between sexes.

Differences in diet were found between athletes and nonathletes (group effect, *p* < 0.05) as well as males and females (sex effect, *p* < 0.05), with no significant sex-by-group interaction. Figure 1 shows Health Canada’s five main food groups, fruits, vegetables, grains, dairy, and meat, and meat alternatives expressed as servings, which are equivalent to one cup per serving. Males reported consuming more servings of grains (F = 5.9, *p* = 0.016), dairy (F = 15.9, *p* < 0.001) and meats and alternatives (F = 9.6, *p* = 0.002) than females (Figure 2A). Athletes reported consuming significantly more servings of fruits (F = 4.6, *p* = 0.034), vegetables (F = 4.0, *p* = 0.047), grains (F = 5.9, *p* = 0.016), dairy (F = 4.4, *p* = 0.038), and meat and meat alternatives (F = 11.8, *p* < 0.001) than nonathletes (Figure 2B).

Table 3 shows a breakdown of the dietary intake in absolute (kcal/day) and relative (kcal/kg/day) terms. There was no sex-by-group interaction in either absolute or relative energy intake. There was a significant main effect for group, with significant differences between athletes and nonathletes for both absolute (517 kcal difference, F = 16.4, *p* < 0.001) and relative (7.8 kcal/kg/day difference, F = 5.7, *p* = 0.019) energy intake. A main effect for sex was found in absolute intake (437 kcal difference, F = 9.6, *p* = 0.002); however, when caloric intake was adjusted for body mass, there were no significant sex differences. Macronutrients are shown as mean values expressed as grams/day (Table 3). There were significant differences between athletes and nonathletes (group effect, *p* < 0.001) and between sexes (sex effect, *p* < 0.001) with no significant interactions. Athletes had a greater intake of carbohydrates (54.5 g/day difference, F = 7.8, *p* = 0.006), fats (21.7 g/day difference, F = 13.6, *p* < 0.001), protein (20.5 g/day difference, F = 11.1, *p* = 0.001), and fiber (5.5 g/day difference, F = 9.7, *p* = 0.002) compared to nonathletes. Similarly, males had a greater intake of carbohydrates (64 g/day difference, F = 11.5, *p* < 0.001), fats (16 g/day difference, F = 7.4, *p* = 0.008), protein (27 g/day difference, F = 18, *p* < 0.001), and fiber (4 g/day difference, F = 4, *p* = 0.047) compared to females. Athletes had higher micronutrient intake, except for copper, compared to nonathletes (group effect, *p* = 0.05) and males had greater micronutrient intake, except for copper, compared to females (sex effect, *p* = 0.05). Except for vitamin D, athletes also had greater vitamin intake compared to nonathletes (group effect, *p* = 0.05). Similarly, males had a significantly greater intake of vitamins, except C and K, compared to females (sex effect, *p* = 0.05).

**Figure 3:**
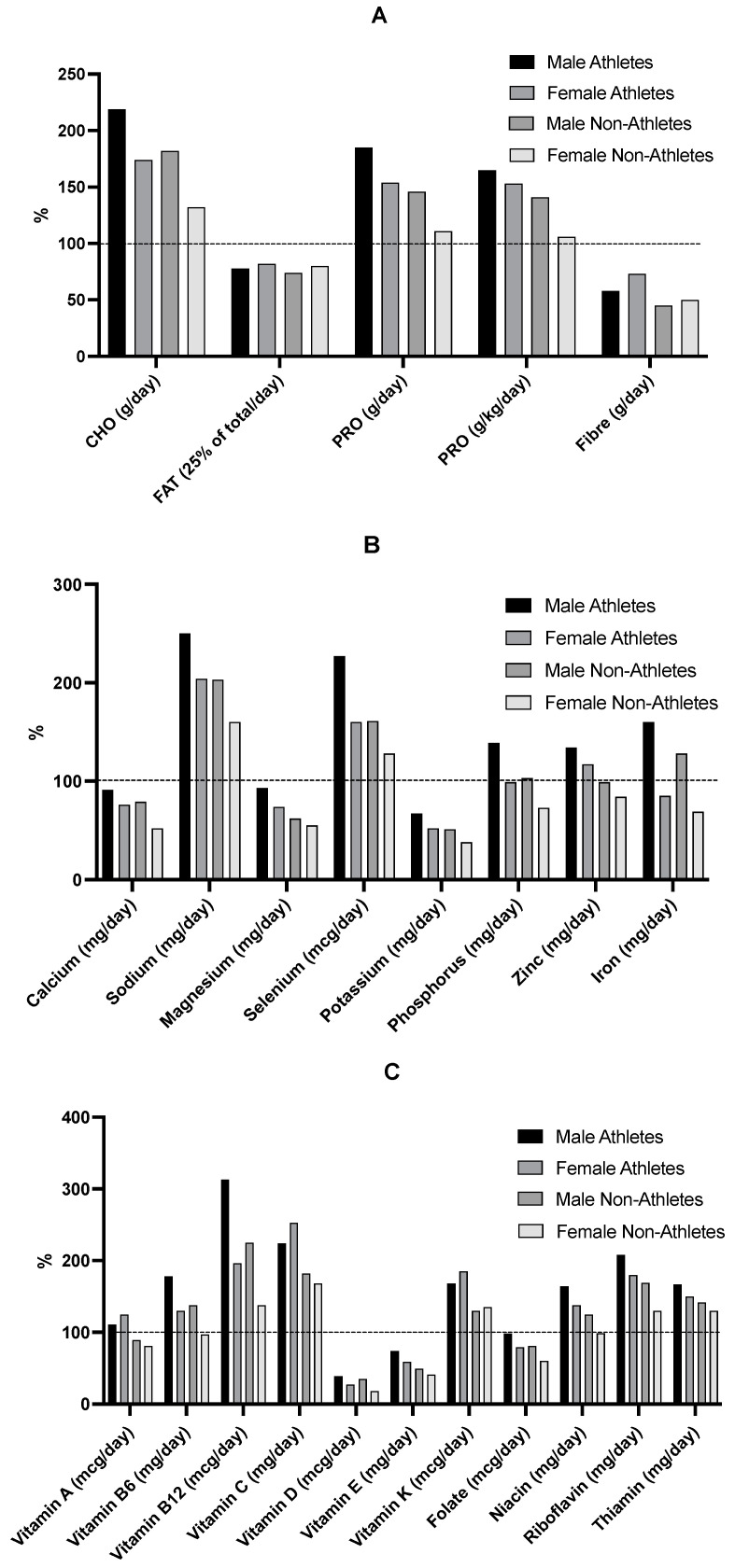
Adequacy of the dietary intake of macronutrients (**A**), micronutrients (**B**), and vitamins (**C**) as percentages of the Canadian recommended dietary intakes (RDI) for adolescent females and males (14–18 years of age). When RDI could not be applied, adequate intake (AI) was used instead. (*n* = 116).

Figure 3 shows the macronutrient, micronutrient, and vitamin intakes as a percentage of adequacy in male and female athletes and male and female nonathletes. Macronutrient RDIs are 130 g/day for carbohydrates, 0.85 g/kg/day of protein (or 52 g/day for males and 46 g/day for females), 38 g/day of fiber for males, and 26 g/day for females [41]. Fat does not have a specific RDI value; however, it is recommended that fats contribute 25–30% of the total energy [41]. Figure 3A shows that all groups were achieving adequate carbohydrate and protein intake. Even when protein intake was adjusted for body mass, intake was still adequate (165%, 153%, 141%, and 106%, respectively). However, none of the groups were meeting the recommendations for fat and fiber (Figure 3A). Figure 3B shows micronutrient intake as a percentage of adequacy in male and female athletes, and male and female nonathletes, with all groups achieving adequate sodium and selenium intake (i.e., 1500 mg/day and 55 mcg/day, respectively) but less calcium, potassium, and magnesium than the RDI for their age and sex (i.e., 1300 mg/day calcium, 4700 mg/day potassium, 410 mg/day magnesium for males, and 360 mg/day magnesium for females) [41]. Given that the RDI for phosphorus is 1250 mg/day and for iron it is 11 mg/day for males and 15 mg/day for females, males (both athletes and nonathletes) were meeting recommendations for phosphorus and iron, but females were below recommendations (Figure 3B). Both male and female athletes met the recommendation for zinc of 11 mg/day and 9 mg/day, respectively [41], but the nonathletes were below the RDI. Lastly, both male and female athletes met almost all vitamin RDIs except for folate, and vitamins D and E. Male nonathletes met most vitamin RDIs, except for vitamin A, vitamins D and E, and folate (Figure 3C). Lastly, female nonathletes were lacking in vitamin intake, only meeting the RDI for riboflavin, thiamin, and vitamins B12, C, and K (Figure 3C).

**Figure 4:**
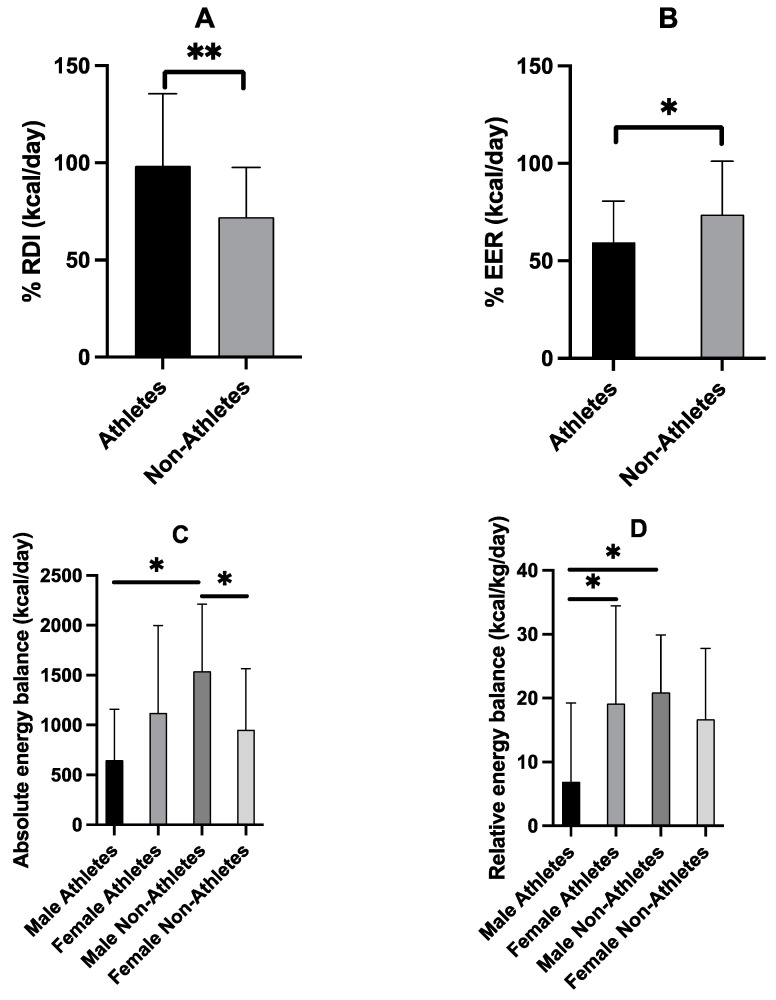
Energy intake as percentage of the Canadian recommended daily intake, i.e., %RDI (**A**) and as percentage of the estimated energy requirements, i.e., %EER (**B**) in athletes (*n* = 58) and nonathletes (*n* = 58). The RDI for adults and youth (ages 13 and older) is 2000 calories a day. EER uses a formula considering participants age, weight, height, and physical activity score to determine the required intake (kcal/day) relative to the individual. Absolute energy balance was then calculated as the difference between energy intake and energy expenditure expressed in kcal/day (**C**) and relative energy balance was calculated as the difference between energy intake and energy expenditure expressed in kcal/kg/day expenditure (**D**). Values are means ± SD. * denotes *p* < 0.05 differences between groups; ** denotes *p* < 0.001 differences between groups.

Figure 4 demonstrates the difference between the energy intake as a percentage of the Canadian RDI, and meeting the energy needs calculated as a percentage of the estimated energy requirements (EER) to maintain an appropriate energy balance. Adults and youth (ages 13 and older) need a minimum average of 2000 calories a day [43]. As shown in Figure 4A, athletes were meeting 98% of RDI (1968 ± 734 kcal/day), while nonathletes were only meeting 72% of RDI (1452 ± 502 kcal/day), and this difference was significant (F = 14.6, *p* < 0.001). However, when intake requirements are made relative to the age, height, body mass, and physical activity score, both athletes and nonathletes were not meeting their estimated EER (Figure 4B). Athletes were only meeting 60% of EER (3322 ± 520 kcal/day), while nonathletes were meeting 74% of EER (2021 ± 510 kcal/day), and this difference was significant (F = 6.9, *p* = 0.001). Finally, a significant sex-by-group interaction was found in both absolute (F = 10.4, *p* = 0.002) and relative (F = 7.9, *p* = 0.006) energy balance. As shown in Figure 4C, male athletes had a significantly lower absolute energy balance than male nonathletes (F = 0.913, *p* = 0.001), and female nonathletes had a significantly lower absolute energy balance than male nonathletes (F = 0.028, *p* = 0.004). Importantly, Figure 4D shows male athletes having significantly lower relative energy balance than female athletes (F = 1.628, *p* = 0.006) and male nonathletes (F = 0.156, *p* = 0.004).

Table 4 illustrates the supplement use amongst athletes and nonathletes. Significantly more athletes reported using performance-related supplements than nonathletes, but there were no differences in the consumption of dietary supplements. Additionally, a greater number of females than males were taking vitamin D (38% versus 14%, respectively; *p* = 0.022) and vitamin C (38% versus 14%, respectively; *p* = 0.022). No other significant differences were found in terms of supplement use.

## 4. Discussion

This is the first study comparing the energy intake, expenditure, and use of supplements between adolescent volleyball athletes and age-matched nonathletic controls. Significant differences were found between athletes and nonathletes, and between male and female athletes. Overall, although the volleyball athletes seemed to match their higher energy expenditure with a greater energy intake, they were only meeting 60% of EER for their age, height, body mass, and physical activity score, while nonathletes were meeting 74% of the EER. Importantly, the relative energy balance of male athletes was lower compared to both female athletes and male nonathletes. In addition, athletes reported consuming more fruits, vegetables, dairy, and grains, but they were still under the RDI of these foods. They also had a higher intake of meats and meat alternatives, as well as fat, carbohydrates, protein, sugar, fibre, vitamin C, calcium, and sodium compared to nonathletes. Similar differences were observed between males and females. In addition, more athletes reported using performance-related supplements than nonathletes, but there were no differences in the consumption of other dietary supplements. These findings support the importance of providing athletes with nutritional guidance during their developmental years, in order to ensure energy needs are met and proper supplement use is introduced in an educated and informed/safe manner to enhance performance in athletes and help them form healthy habits.

Although the higher energy expenditure of athletes compared to that of nonathletes is to be expected, it is important to note that nonathletes were not meeting the recommended 60 min of MVPA for 14–18-year-old Canadians [44]. There were also significant differences between male and female athletes, with males almost doubling the relative calories expended and minutes of MVPA compared to females. This difference in sexes among volleyball athletes is in contrast with previous findings in distance runners, where relative expenditure adjusted to body mass showed no differences between males and females [45]. However, similar to our findings, other studies have also reported greater total energy expenditure in adolescent male athletes (basketball and ballet) compared to female athletes [46,47].

As of 2019, the food serving recommendations for Canadian youth shifted from providing a serving recommendation to instead providing the type of food from each group that should be favoured (i.e., dark leafy greens). Thus, for the purpose of our study, we compared the 2007 Canada’s Food Guide recommendation to the participants’ actual serving intake [48]. Daily fruit and vegetable servings were not meeting the recommended seven servings/day for females and eight servings/day for males aged 14–18 years. Similarly, although the athletes did consume more fruits and vegetables than the nonathletes, both groups were not meeting the recommendations [48], even when combining the two categories together. These results agree with previous studies in adolescents, reporting both sexes not meeting the recommendations for fruits, vegetables, and grains [44,49]. Furthermore, Canada’s Food Guide does not take into account high levels of physical activity, so many recommendations may need to be higher for young athletes to make up for their greater energy requirements [25]. Based on our findings, along with past research, young athletes need to be encouraged to consume more grains, dairy, as well as vegetables and fruits [25]. On the other hand, our participants met the recommendation for meats and meat alternatives, with an expected greater intake in males compared to females and in athletes compared to nonathletes. This finding contradicts previous reports showing inadequate intake of meat and meat alternatives in athletes and nonathletes [25,44]. It is therefore likely that our volleyball players were making up for the lack of fruits, vegetables, grain, and dairy intake with an increased intake of meats and meat alternatives. This further supports the findings of greater-than-average protein intake in athletes and nonathletes, regardless of sex.

The diet of an adolescent is typically energy-dense and lacking in proper nutrients [50]; however, studies have found that adolescent athletes show improved intake and diet quality compared to adolescent nonathletes [51,52,53]. Energy deficiency is common in athletes [54], with energy intake not in balance with the energy demands of the sport. The recommended caloric intake in males and females 13 years and older is a minimum of 2000 calories a day [48]. Our study shows that athletes and nonathletes are not meeting this recommendation and are consuming fewer calories than athletes from previous studies of similar age and training status [55,56,57,58]. We also compared the reported dietary intakes for macronutrients to those recommended by Health Canada for each sex [48]. While athletes reported a greater daily intake of carbohydrates than nonathletes, it is difficult to determine whether this intake is appropriate for the demands of the sport. Previous research has shown inadequate carbohydrate intake in young athletes [57,58,59]. Indeed, the dietary fibre intakes reported herein did not meet recommendations, suggesting that information on the quantity and quality of carbohydrates and fibre-rich foods should be provided to this age group. Likewise, our participants reported low fat intake compared to the recommended 25–30% of total calories [60]. Reported intakes of fat have varied across studies, with low to high intakes in adolescent athletes [55,56,57,58,61]. Conversely, if a young athlete was struggling to meet caloric needs, healthy fats would be the most energy-dense option. Average protein intakes exceeded the upper recommendation in all groups, suggesting that this is not a nutrient of concern for young Canadian athletes; this is a finding supported by others [55,57,59,62]. It has been suggested that protein intakes are generally adequate or excessive because protein is overvalued among coaches and athletes [63,64,65].

Athletes met most micronutrient and vitamin recommendations for adolescents, even when some nonathletes did not. Notably, however, females tended to have lower intakes relative to males, highlighting this group as being at higher risk of nutritional deficiencies. Such deficiencies are particularly problematic for nutrients such as calcium, phosphorus, and vitamin D, which are important for bone accrual in this age group [56], and this is even more concerning when coupled with energy deficiency [66]. This is not the only study to examine adolescent athlete micronutrient and vitamin intake and find deficiencies in phosphorus, calcium, vitamin D, folate, iron, and potassium, particularly in females [25]. The dangers of not meeting the recommendations for these nutrients, especially for athletes, are increased risks of low bone mineral density, anemia, fatigue, and suboptimal athletic performance [57,67]. Addressing this insufficiency could be carried out by addressing the low dairy servings intake, and providing nutritional knowledge regarding the benefit of dairy products on bone health.

Previous studies investigating the energy balance of adolescent athletes have highlighted the importance of meeting and maintaining energy needs, especially in female athletes, and the increasing prevalence of the female-athletes triad [68]. The female-athletes triad, although complex and multifactorial, has been shown to be greatly associated with energy balance [69,70], with relative energy deficiency in sport (RED-S) resulting in impaired physiological function including, but not limited to, metabolic rate, menstrual function, bone health, immunity, protein synthesis, and cardiovascular health [71]. RED-S has also been shown not only in female athletes but also male athletes [71,72]. Our study showed that both RDI and EER were not being met by both athletes and nonathletes, with athletes only meeting 60% of their higher EER needs to cover the energy demands of the sport. This once again highlights the importance of improving the nutritional knowledge of young athletes, as it relates to bone health, energy availability, and in females menstrual function [71]. Athletes who chronically suffer from low energy availability are at increased risk for developing nutrient deficiencies such as anemia, and chronic fatigue, and are at increased risk of infections and illnesses, all of which have the potential to harm their health and performance [73].

Regardless of age, sex, or training status, sport nutrition professionals clearly state that athletes should not use dietary supplements to compensate for a poor diet but rather consume whole foods to meet their nutrient needs. Only when instructed by a medical professional should athletes rely on supplements [61,67]. However, the recent trend of protein supplementation among athletes and coaches has led to an increase in protein supplement intake, which appears to be unnecessary given the high dietary protein intake [61]. Sports drinks could be justified during longer training sessions, as insufficient carbohydrate intake during exercise has been identified as a weakness in some young athletes [62]. However, our athletes’ carbohydrate consumption was sufficient, so greater emphasis should be placed on quality and not quantity. The importance of proper supplementation knowledge in adolescents is to establish healthy habits earlier in life, as supplement consumption increases with age [74]. However, as the athletes age and compete at a higher level, the level of competition becomes a factor, as these products are often viewed as performance-enhancing. Evidence has shown that Canadian male athletes are more likely to use supplements for overall athletic performance than their female counterparts [58,74]. Our own results are limited regarding males; however, we found no sex differences in performance-related supplement use.

This study has several limitations. The main limitation is that our sample consisted heavily of females, and, thus, cannot be considered representative of the general volleyball population. Thus, the results of this study cannot be generalized to both male and female volleyball athletes, and should be interpreted with caution. In addition, the results of this study are based on self-reported data, and, thus, are subject to recall error and social desirability bias. Likewise, energy expenditure was not measured physiologically. Future studies should use standard physiological methods (e.g., direct calorimetry) to measure the metabolic rates of adolescent athletes and nonathletes.

## 5. Conclusions

Overall, when compared to nonathletic controls, both male and female adolescent volleyball athletes were found to match their higher energy expenditure with greater dietary energy intake; however, all adolescents were below the estimated energy requirements, a finding more profound among the volleyball athletes. These findings support the importance of providing adolescent athletes with nutritional guidance during their developmental years, in order to ensure that their nutrition is healthy and sufficient to support both their performance and growth needs.

## Figures and Tables

**Table 1:** Demographic characteristics of participants.

Demographics	Athletes		Nonathletes	
	*n*	%	*n*	%
	58	-	58	-
**Age (years)**				
14	23	40	15	26
15	15	26	17	29
16	14	24	14	24
17	6	10	12	21
**Sex**				
Male	15	26	13	22
Female	43	74	45	78
**Household Income**				
<$25,000/year	7	12	6	10
$25,000–$50,000/year	17	29	6	10
$50,000–$100,000/year	8	14	18	31
$100,000–$200,000/year	13	22	11	19
>$200,000	9	16	14	24
No Answer	4	7	3	5
**Training (years)**				
1–2	12	21	-	-
3–5	34	58	-	-
6+	12	21	-	-
**Training volume (hours/week)**				
6–7	33	14	-	-
8–9	10	43	-	-
10+	15	14	-	-

**Table 2:** Physical characteristics of male and female athletes and nonathletes.

	Male Athletes (*n* = 15)	Male Nonathletes (*n* = 13)	Female Athletes (*n* = 43)	Female Nonathletes (*n* = 45)
Height (cm)	179.5 ± 9	177 ± 9	168 ± 8	168 ± 8
Weight (kg)	68.3 ± 10	66.3 ± 18	57.5 ± 7	58 ± 9
BMI (kg/m^2^)	21.1 ± 1.5	21.3 ± 4.3	20.4 ± 2.1	20.6 ± 2.0
BMI Classification (*n* [%])				
Underweight	1 [7%]	4 [30%]	10 [23%]	10 [22%]
Normal Weight	14 [93%]	7 [54%]	32 [74%]	32 [71%]
Overweight	0 [0%]	1 [8%]	1 [2%]	3 [7%]
Obesity	0 [0%]	1 [8%]	0 [0%]	0 [0%]

**Table 3:** Caloric intake, macronutrients, micronutrients, and vitamins are shown in athletes and nonathletes, and males and females. (*n* = 116). Values are means ± SD. Bolded ***p*** values are significant.

Measure	Athletes (*n* = 58)	Nonathletes (*n* = 58)	*p*	Males (*n* = 28)	Females (*n* = 88)	*p*
**Caloric Intake**						
Absolute (kcal/day)	1968 ± 734	1451 ± 502	<0.001	2041 ± 739	1604 ± 625	0.002
Relative (kcal/kg/day)	33.5 ± 13	25.7 ± 11	0.019	32.6 ± 14	28.6 ± 13	0.159
**Macronutrients**						
Carbohydrates (g/day)	241 ± 99	186.5 ± 73	**0.006**	262 ± 111	198 ± 79	**<0.001**
Fats (g/day)	81 ± 29	59.3 ± 23	**<0.001**	82 ± 27	66 ± 28	**0.008**
Protein (g/day)	77 ± 33	56.5 ± 25	**0.001**	87 ± 35	60 ± 26	**<0.001**
Fibre (g/day)	19.6 ± 9	14.1 ± 6	**0.002**	20 ± 10	16 ± 7	**0.047**
**Micronutrients**						
Calcium (mg/day)	1034 ± 462	759 ± 371	**0.011**	1113 ± 391	828 ± 433	**0.002**
Iron (mg/day)	14.1 ± 6	11.1 ± 5	**0.015**	16 ± 8	11.5 ± 4.6	**<0.001**
Phosphorus (mg/day)	1368 ± 565	1001 ± 427	**<0.001**	1531 ± 610	1071 ± 452	**<0.001**
Magnesium (mg/day)	297 ± 146	212 ± 84	**<0.001**	322.9 ± 166	232 ± 102	**<0.001**
Potassium (mg/day)	2631 ± 1002	1936 ± 718	**<0.001**	2822 ± 1017	2106 ± 840	**<0.001**
Sodium (mg/day)	3236 ± 1205	2542 ± 1020	**0.005**	3421 ± 1327	2719 ± 1061	**0.006**
Zinc (mg/day)	11.6 ± 6	8.3 ± 4	**0.001**	13 ± 6	9 ± 4.3	**<0.001**
Selenium (mcg/day)	97.7 ± 45	74.6 ± 33	**0.001**	107.8 ± 51	79 ± 34	**0.001**
Copper (mg/day)	1.74 ± 2.7	0.98 ± 0.4	0.114	1.46 ± 0.7	1.32 ± 2.2	0.823
**Vitamins**						
Vitamin A (mcg/day)	905 ± 441	620 ± 296	**0.002**	904 ± 345	718 ± 408	**0.031**
Vitamin B6 (mg/day)	1.8 ± 0.87	1.3 ± 0.5	**0.002**	2.1 ± 0.97	1.4 ± 0.59	**<0.001**
Vitamin B12 (mcg/day)	5.4 ± 3	3.8 ± 1.9	**0.001**	6.5 ± 2.8	3.99 ± 2.5	**<0.001**
Vitamin C (mg/day)	166 ± 92	115 ± 71	**0.018**	154 ± 107	136 ± 79	0.392
Vitamin D (mcg/day)	4.9 ± 2.8	3.3 ± 2.1	0.062	5.5 ± 2.7	3.4 ± 2.3	**<0.001**
Vitamin E (mg/day)	9.5 ± 4.6	6.4 ± 2.5	**<0.001**	9.5 ± 4.9	7.5 ± 3.6	**0.032**
Vitamin K (mcg/day)	136 ± 62	100 ± 47	**0.009**	113 ± 55	120 ± 59	0.493
Folate (mcg/day)	334 ± 128	258 ± 100	**0.004**	360 ± 149	275 ± 102	**<0.001**
Niacin (mg/day)	21.1 ± 10	15.2 ± 6	**<0.001**	23.3 ± 11.3	16.5 ± 7.2	**<0.001**
Riboflavin (mg/day)	2.0 ± 0.9	1.54 ± 0.7	**0.002**	2.48 ± 11.3	1.57 ± 0.7	**<0.001**
Thiamin (mg/day)	1.7 ± 0.61	1.4 ± 0.6	**0.045**	1.9 ± 0.69	1.4 ± 0.56	**<0.001**

**Table 4:** Number of athletes and nonathletes using performance and dietary supplements and mean intake of each supplement used.

Supplement	Athletes (*n* = 58)			Nonathletes (*n* = 58)			
	*n*	%	Mean Intake	*n*	%	Mean Intake	χ^2^ (*p*)
**Performance**							
Sport drinks	54	93	106 ± 179 *	42	72%	45 ± 65 *	0.036
Protein drinks/liquid meals	4	7	5.2 ± 2.1	2	3%	10.5 ± 9	0.402
Protein bars	23	40	10.8 ± 17.0	15	26%	7.1 ± 9	0.002
**Dietary**							
Vitamin A (mcg/day)	18	31	1509 ± 978	17	29	1599 ± 1294	0.821
Lutein + Zeaxanthin	0	0	0 ± 0	1	2	143	0.315
Vitamin D (mcg/day)	25	43	22.6 ± 13	22	38	22 ± 19	0.550
Vitmain E (mg/day)	17	29	23.7 ± 17	16	28	28.3 ± 23	0.816
Vitamin K (mcg/day)	14	24	19.1 ± 9	12	21	18.0 ± 7	0.590
Thiamin (mg/day)	16	28	1.8 ± 1.5	15	26	2.0 ± 1	0.809
Riboflavin (mg/day)	16	28	2.0 ± 1.5	15	26	2.2 ± 1.2	0.809
Niacin (mg/day)	16	28	19.6 ± 12	15	26	29.5 ± 15	0.809
Vitamin B6 (mg/day)	17	29	34.4 ± 47	15	26	43.8 ± 45	0.636
Folate (mcg/day)	17	29	536 ±406	15	26	797 ± 403	0.636
Vitamin B12 (mcg/day)	19	33	312 ± 457	17	29	425 ± 458	0.656
Vitamin C (mg/day)	26	45	381 ± 304	21	36	335 ± 283	0.319
Calcium (mg/day)	18	31	195 ± 145	18	31	220 ± 131	1.000
Copper (mg/day)	16	28	0.9 ± 0.8	15	26	1.3 ± 0.8	0.809
Iron (mg/day)	19	33	35.4 ± 30	16	28	40.6 ± 22	0.498
Magnesium (mg/day)	16	28	48.5 ± 30	15	26	53.1 ± 34	0.809
Selenium (mcg/day)	16	28	51.6 ± 31	15	26	55.9 ± 36	0.809
Zinc (mg/day)	17	29	14.9 ± 14	16	28	14.5 ± 7	0.816
Alpha-linolenic acid	2	3	294.6 ± 64	0	0	0 ± 0	0.154
Oleic acid	12	21	186.7 ± 110	10	17	202 ± 136	0.542
Omega-3 fatty acids	12	21	273.2 ± 125	12	21	184 ± 176	1.000
Omega-6 fatty acids	12	21	64.0 ± 100	12	21	6.0 ± 3	1.000
Eicosapentaenoic acid	10	17	123.7 ± 70	10	17	116 ± 79	1.000
Docosahexaenoic acid	10	17	85.8 ± 48	10	17	80.7 ± 55	1.000
Fiber (g/day)	0	0	0 ± 0	8	14	0.6 ± 0.6	0.315

* denotes significant differences between groups in the mean intake (*p* < 0.05).

## Data Availability

Due to ethical restrictions, the data presented in this study are available to researchers eligible under the Research Ethics Board rules on request from the corresponding author.

## References

[1] Ortega F.B., Ruiz J.R., Castillo M.J., Sjöström M. (2008). Physical fitness in childhood and adolescence: A powerful marker of health. Int. J. Obes..

[2] Huang C., Memon A.R., Yan J., Lin Y., Chen S.T. (2021). The Associations of Active Travel to School with Physical Activity and Screen Time Among Adolescents: Do Individual and Parental Characteristics Matter?. Front. Public Health.

[3] Verhagen E.A., Van der Beek A.J., Bouter L.M., Bahr R.M., Van Mechelen W. (2004). A one season prospective cohort study of volleyball injuries. Br. J. Sport. Med..

[4] Tipton K.D. (2015). Nutritional support for exercise-induced injuries. Sport. Med..

[5] Kreider R.B., Wilborn C.D., Taylor L., Campbell B., Almada A.L., Collins R., Cooke M., Earnest C.P., Greenwood M., Kalman D.S. (2010). ISSN exercise & sport nutrition review: Research & recommendations. J. Int. Soc. Sports Nutr..

[6] Wenzel R.K., Valliant M.W., Chang Y., Bomba A.K., Lambert L.G. (2012). Dietary Assessment and Education Improves Body Composition and Diet in NCAA Female Volleyball Players. Top. Clin. Nutr..

[7] Caparello G., Galluccio A., Ceraudo F., Pecorella C., Buzzanca F., Cuccomarino F., Bonofiglio D., Avolio E. (2023). Evaluation of Body Composition Changes by Bioelectrical Impedance Vector Analysis in Volleyball Athletes Following Mediterranean Diet Recommendations during Italian Championship: A Pilot Study. Appl. Sci..

[8] Kerksick C.M., Wilborn C.D., Roberts M.D., Smith-Ryan A., Kleiner S.M., Jäger R., Collins R., Cooke M., Davis J.N., Galvan E. (2018). ISSN Exercise & Sports Nutrition Review Update: Research & Recommendations. J. Int. Soc. Sports Nutr..

[9] Forbes G.B. (1981). Body composition in adolescence. Prog. Clin. Biol. Res..

[10] Mielgo-Ayuso J., Zourdos M.C., Calleja-González J., Urdampilleta A., Ostojic S.M. (2015). Dietary intake habits and controlled training on body composition and strength in elite female volleyball players during the season. Appl. Physiol Nutr. Metab..

[11] Holway F.E., Spriet L.L. (2011). Sport-Specific Nutrition: Practical Strategies for Team Sports. J. Sports Sci..

[12] Burke L.M., Hawley J.A., Wong S.H.S., Jeukendrup A.E. (2011). Carbohydrates for Training and Competition. J. Sport. Sci..

[13] Phillips S.M., Van Loon L.J. (2011). Dietary protein for athletes: From requirements to optimum adaptation. J. Sports Sci..

[14] Rodríguez N.R., Di Marco N.M., Langley S. (2009). American college of sports medicine position stand nutrition and athletic performance. Med. Sci. Sports Exerc..

[15] Maughan R.J., Burke L.M., Dvorak J., Larson-Meyer D.E., Peeling P., Phillips S.M., Rawson E.S., Walsh N.P., Garthe I., Geyer H. (2018). IOC Consensus Statement: Dietary Supplements and the High-Performance Athlete. Int. J. Sport Nutr. Exerc. Metab..

[16] Stellingwerff T., Cox G.R. (2014). Systematic review: Carbohydrate supplementation on exercise performance or capacity of varying durations. Appl. Physiol. Nutr. Metab..

[17] Peake J.M., Neubauer O., Walsh N.P., Simpson R.J. (2017). Recovery of the immune system after exercise. J. Appl. Physiol..

[18] Kozirok W., Babicz-Zielińska E., Krzebietke B. (2013). An Assessment of the Consumption of Dietary Supplements by Players of Selected Sports. Pol. J. Sport Tour..

[19] Mariscal-Arcas M., Monteagudo C., Hernandez-Elizondo J., Benhammou S., Lorenzo L., Olea-Serrano F. (2015). Differences in food intake and nutritional habits between Spanish adolescents who engage in ski activity and those who do not. Nutr. Hosp..

[20] McGuine T.A., Sullivan J.C., Bernhardt D.T. (2001). Creatine supplementation in high school football players. Clin. J. Sport Med..

[21] Wiens K., Erdman K.A., Stadnyk M., Parnell J.A. (2014). Dietary Supplement Usage, Motivation, and Education in Young Canadian Athletes. Int. J. Sport Nutr. Exerc. Metab..

[22] Erdman K.A., Fung T.S., Doyle-Baker P.K., Verhoef M.J., Reimer R.A. (2007). Dietary supplementation of high-performance Canadian athletes by age and gender. Clin. J. Sport Med..

[23] Erdman K.A., Fung T.S., Reimer R.A. (2006). Influence of Performance Level on Dietary Supplementation in Elite Canadian Athletes. Med. Sci. Sport. Exerc..

[24] Giannopoulou I., Noutsos K., Apostolidis N., Bayios I., Nassis G.P. (2013). Performance level affects the dietary supplement intake of both individual and team sports athletes. J. Sport. Sci. Med..

[25] Parnell J.A., Wiens K.P., Erdman K.A. (2016). Dietary Intakes and Supplement Use in Pre-Adolescent and Adolescent Canadian Athletes. Nutrients.

[26] Sato A., Kamei A., Kamihigashi E., Dohi M., Komatsu Y., Akama T., Kawahara T. (2012). Use of supplements by young elite Japanese athletes participating in the 2010 youth Olympic games in Singapore. Clin. J. Sport Med..

[27] Ziegler P.J., Nelson J.A., Jonnalagadda S.S. (2003). Use of dietary supplements by elite figure skaters. Int. J. Sport Nutr. Exerc. Metab..

[28] Huang S.-H.S., Johnson K., Pipe A.L. (2006). The use of dietary supplements and medications by Canadian athletes at the Atlanta and Sydney Olympic Games. Clin. J. Sport Med..

[29] McGuine T.A., Sullivan J.C., Bernhardt D.A. (2002). Creatine supplementation in Wisconsin high school athletes. WMJ-MADISON-.

[30] Scofield D.E., Unruh S. (2006). Dietary supplement use among adolescent athletes in central Nebraska and their sources of information. J. Strength Cond. Res..

[31] Solheim S.A., Nordsborg N.B., Ritz C., Berget J., Kristensen A.H., Mørkeberg J. (2017). Use of nutritional supplements by Danish elite athletes and fitness customers. Scand. J. Med. Sci. Sport..

[32] Diehl K., Thiel A., Zipfel S., Mayer J., Schnell A., Schneider S. (2012). Elite adolescent athletes’ use of dietary supplements: Characteristics, opinions, and sources of supply and information. Int. J. Sport Nutr. Exerc. Metab..

[33] Braun H., Koehler K., Geyer H., Kleinert J., Mester J., Schänzer W. (2009). Dietary Supplement Use among Elite Young German Athletes. Int. J. Sport Nutr. Exerc. Metab..

[34] de Silva A., Samarasinghe Y., Senanayake D., Lanerolle P. (2010). Dietary supplement intake in national-level Sri Lankan athletes. Int. J. Sport Nutr. Exerc. Metab..

[35] Metzl J.D., Small E., Levine S.R., Gershel J.C. (2001). Creatine Use Among Young Athletes. Pediatrics.

[36] Suzic Lazic J., Dikic N., Radivojevic N., Mazic S., Radovanovic D., Mitrovic N., Lazic M., Zivanic S., Suzic S. (2011). Dietary supplements and medications in elite sport—Polypharmacy or real need?: Dietary supplement and medication use in elite sport. Scand. J. Med. Sci. Sport..

[37] von Elm E.B., Altman D.G., Egger M., Pocock S.J., Gøtzsche P.C., Vandenbroucke J.P. (2007). The Strengthening the Reporting of Observational Studies in Epidemiology (STROBE) statement: Guidelines for reporting observational studies. PLoS Med..

[38] Akhtar-Danesh N., Dehghan M., Merchant A.T., ARainey J. (2008). Validity of self-reported height and weight for measuring prevalence of obesity. Open Med. Peer-Rev. Indep. Open-Access J..

[39] Block G., Woods M., Potosky A., Clifford C. (1990). Validation of a self-administered diet history questionnaire using multiple diet records. J. Clin. Epidemiol..

[40] Boucher B., Cotterchio M., Kreiger N., Nadalin V., Block T., Block G. (2006). Validity and reliability of the Block98 food-frequency questionnaire in a sample of Canadian women. Public Health Nutr..

[41] (2014). US/Canadian recommended dietary allowances and acceptable intakes. A Dictionary of Food and Nutrition.

[42] Kang J. (2018). Nutrition and Metabolism in Sports, Exercise and Health. Guidelines for Designing a Healthy and Competitive Diet.

[43] Yon B.A., Johnson R.K. (2005). US and Canadian Dietary Reference Intakes (DRIs) for the macronutrients, energy and physical activity. Nutr. Bull..

[44] Storey K.E., Forbes L.E., Fraser S.N., Spence J.C., Plotnikoff R.C., Raine K.D., Hanning R.M., McCargar L.J. (2009). Diet quality, nutrition and physical activity among adolescents: The Web-SPAN (Web-Survey of Physical Activity and Nutrition) project. Public Health Nutr..

[45] Eisenmann J., Wickel E. (2007). Estimated Energy Expenditure and Physical Activity Patterns of Adolescent Distance Runners. Int. J. Sport Nutr. Exerc. Metab..

[46] Katzmarzyk P.T., Malina R.M. (1998). Contribution of Organized Sports Participation to Estimated Daily Energy Expenditure in Youth. Pediatr. Exerc. Sci..

[47] Hirsch N.M. (2002). Total daily energy expenditure and physical activity patterns in university ballet dancers. J. Dance Med. Sci..

[48] Health Canada (2007). Eating Well with Canada’s Food Guide: A Resource for Educators and Communicators.

[49] Rossiter M.D., Evers S.E., Pender A.C. (2012). Adolescents’ diets do not comply with 2007 Canada’s food guide recommendations. Appetite.

[50] Diethelm K., Jankovic N., Moreno L.A., Huybrechts I., De Henauw S., De Vriendt T., González-Gross M., Leclercq C., Gottrand F., Gilbert C.C. (2012). Food intake of European adolescents in the light of different food-based dietary guidelines: Results of the HELENA (Healthy Lifestyle in Europe by Nutrition in Adolescence) Study. Public Health Nutr..

[51] Garcin M., Doussot L., Mille-Hamard L., Billat V. (2009). Athletes’ dietary intake was closer to French RDA’s than those of young sedentary counterparts. Nutr. Res..

[52] Cavadini C., Decarli B., Grin J., Narring F., Michaud P.A. (2000). Food habits and sport activity during adolescence: Differences between athletic and non-athletic teenagers in Switzerland. Eur. J. Clin. Nutr..

[53] Croll J.K., Neumark-Sztainer D., Story M., Wall M., Perry C., Harnack L. (2006). Adolescents Involved in Weight-Related and Power Team Sports Have Better Eating Patterns and Nutrient Intakes than Non−Sport-Involved Adolescents. J. Am. Diet. Assoc..

[54] Noll M., de Mendonça C.R., de Souza Rosa L.P., Silveira E.A. (2017). Determinants of eating patterns and nutrient intake among adolescent athletes: A systematic review. Nutr. J..

[55] Aerenhouts D., Deriemaeker P., Hebbelinck M., Clarys P. (2011). Energy and macronutrient intake in adolescent sprint athletes: A follow-up study. J. Sport. Sci..

[56] Dwyer J., Eisenberg A., Prelack K., Song W.O., Sonneville K., Ziegler P. (2012). Eating attitudes and food intakes of elite adolescent female figure skaters: A cross sectional study. J. Int. Soc. Sport. Nutr..

[57] Gibson J.C., Stuart-Hill L., Martin S., Gaul C. (2011). Nutrition status of junior elite Canadian female soccer athletes. Int. J. Sport Nutr. Exerc. Metab..

[58] Papadopoulou S.K., Papadopoulou S.D., Gallos G.K. (2002). Macro- and micro-nutrient intake of adolescent Greek female volleyball players. Int. J. Sport Nutr. Exerc. Metab..

[59] Nikic M., Pedišic Ž., Šatalic Z., Jakovljevic S., Venus D. (2014). Adequacy of nutrient intakes in elite junior basketball players. Int. J. Sport Nutr. Exerc. Metab..

[60] Trumbo P., Schlicker S., Yates A.A., Poos M. (2002). Dietary reference intakes for energy, carbohydrate, fiber, fat, fatty acids, cholesterol, protein, and amino acids. J. Am. Diet. Assoc..

[61] Desbrow B., McCormack J., Burke L.M., Cox G.R., Fallon K., Hislop M., Logan R., Marino N., Sawyer S.M., Shaw G. (2014). Sports Dietitians Australia position statement: Sports nutrition for the adolescent athlete. Int. J. Sport Nutr. Exerc. Metab..

[62] Baker L.B., Heaton L.E., Nuccio R.P., Stein K.W. (2014). Dietitian-observed macronutrient intakes of young skill and team-sport athletes: Adequacy of pre, during, and postexercise nutrition. Int. J. Sport Nutr. Exerc. Metab..

[63] Juzwiak C.R., Amancio OM S., Vitalle MS S., Pinheiro M.M., Szejnfeld V.L. (2008). Body composition and nutritional profile of male adolescent tennis players. J. Sport. Sci..

[64] Mazzulla M., Volterman K.A., Packer J.E., Wooding D.J., Brooks J.C., Kato H., Moore D.R. (2018). Whole-body net protein balance plateaus in response to increasing protein intakes during post-exercise recovery in adults and adolescents. Nutr. Metab..

[65] Juzwiak C., Ancona-Lopez F. (2004). Evaluation of nutrition knowledge and dietary recommendations by coaches of adolescent Brazilian athletes. Int. J. Sport Nutr. Exerc. Metab..

[66] Nichols J.F., Rauh M.J., Barrack M.T., Barkai H.-S. (2007). Bone mineral density in female high school athletes: Interactions of menstrual function and type of mechanical loading. Bone.

[67] (2000). American Dietetic Association, Dietitians of Canada, and the American College of Sports Medicine. Position Statement. J. Am. Diet. Assoc..

[68] Burrows M., Bird S. (2000). The Physiology of the Highly Trained Female Endurance Runner. Sport. Med..

[69] Kazis K., Iglesias E. (2003). The female athlete triad. Adolesc. Med. Clin..

[70] Otis C.L., Drinkwater B., Johnson M., Loucks A., Wilmore J. (1997). American College of Sports Medicine position stand. The Female Athlete Triad. Med. Sci. Sport. Exerc..

[71] Mountjoy M., Sundgot-Borgen J., Burke L., Carter S., Constantini N., Lebrun C., Meyer N., Sherman R., Steffen K., Budgett R. (2014). The IOC consensus statement: Beyond the Female Athlete Triad—Relative Energy Deficiency in Sport (RED-S). Br. J. Sport. Med..

[72] Sundgot-Borgen J., Meyer N.L., Lohman T.G., Ackland T.R., Maughan R.J., Stewart A.D., Müller W. (2013). How to minimise the health risks to athletes who compete in weight-sensitive sports review and position statement on behalf of the Ad Hoc Research Working Group on Body Composition, Health and Performance, under the auspices of the IOC Medical Commission. Br. J. Sport. Med..

[73] Nattiv A., Loucks A.B., Manore M.M., Sanborn C.F., Sundgot-Borgen J., Warren M.P. (2007). The female athlete triad. Med. Sci. Sport. Exerc..

[74] Evans M.W., Ndetan H., Perko M., Williams R., Walker C. (2012). Response to: Commentary to “Dietary Supplement Use by Children and Adolescents in the United States to Enhance Sport Performance: Results of the National Health Interview Survey.”. J. Prim. Prev..

